# Effective Systemic Treatment of Choroidal Metastases NSCLC With Surgery After Crizotinib: A Case Report

**DOI:** 10.3389/fonc.2022.789941

**Published:** 2022-03-31

**Authors:** Shilan Liu, Xiao Liu, Ting Wang, Chunhua Zeng, Baichen Ren, Xiaodan Yu, Min Xu, Wenjuan Li, Zhihui Qiao, Chuanyun You, Qinghui Yang, Mei Chen

**Affiliations:** ^1^ Department of Respiratory and Critical Care Medicine, Chengdu Fifth People’s Hospital, Chengdu, China; ^2^ Department of Respiratory and Critical Care Medicine, The First Affiliated Hospital of Chongqing Medical University, Chongqing, China; ^3^ Lung Cancer Center, West China Hospital of Sichuan University, Chengdu, China

**Keywords:** choroidal metastasis, non-small cell lung carcinoma, EML4-ALK translocation, surgery, oligometastases, crizotinib

## Abstract

Choroidal metastasis as an initial presenting feature of lung cancer with EML4-ALK translocation is exceedingly rare and greatly impacts patient quality of life (QOL). There are no recommended treatments for such patients, and palliative care remains limited. It is unclear whether surgical resection of primary pulmonary lesions, systemic antitumor therapy, targeted therapy, or localized ocular therapy are effective in treating choroidal metastases in EML4-ALK rearranged oligometastatic non-small cell lung cancer (NSCLC). Here, we present the case of choroidal metastases secondary to lung cancer and EML4-ALK translocation in a 57-year-old woman who firstly underwent resection of lung lesions followed by oral administration of crizotinib without local treatment or systemic chemotherapy. Since then she had a rapid and complete response to crizotinib with 27 months of progression-free survival.

## Introduction

Choroidal metastasis as an initial presenting feature of lung cancer is rare, accompanied with EML4-ALK mutation is exceedingly rarer (1). Furthermore, data addressing the optimal therapeutic strategy is unclear (2). We presented a case report of a 57-year-old woman affected by ALK rearranged NSCLC with a choroidal metastasis, a rare event. The patient presented with oligometastatic disease that was initially suitable for surgical treatment of all sites of disease. The patient underwent surgery on the primary tumor but, as she refused to consent to ocular enucleation, systemic treatment with the TKI crizotinib was undertaken, with clinical benefit and optimal and durable response.

## Case Report

**Figure 1:**
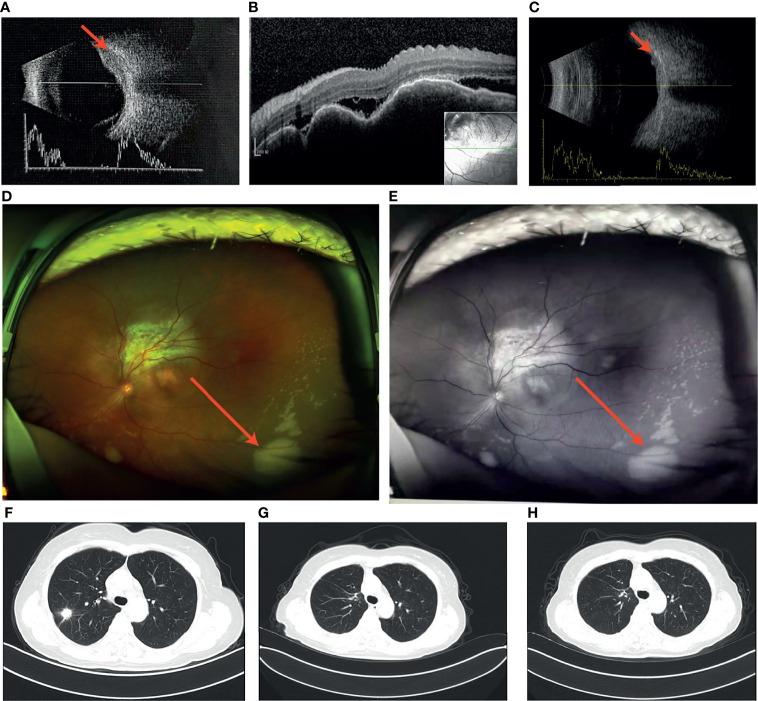
**(A)** Ultrasound revealed the initial dome-like choroid lesion with the height 2.5 mm: **(B)** Optical coherence tomography (OCT) demonstrated a plateau-like elevation of choroid with underlying sub retinal fluid; **(C)** The height of the lesion reduced to 0.75 mm 18 months after crizotinib therapy; **(D,E)** Left fundus image revealed a large dome-shaped amelanotic tumor; **(F)** A nodule in the right upper lung; **(G)** Regression of the primary lesion 2 month after surgery; **(H)** No progression of malignancy in the 18th month of crizotinib therapy.

A 57-year-old Chinese woman, non-smoker, with a 3-month history of reduced left eye visual acuity (20/25) visited an ophthalmological clinic to be examined. She initially manifested eye watering, foreign body sensation, photophobia, blurred vision and swelling of the eyes. Ultrasonographic examination demonstrated an initial dome-shaped lesion with high internal reflectivity and a thickness of approximately 2.5 mm (**Figure 1A**). Optical coherence tomography demonstrated a plateau-like elevation resulting from multiple, non-primitive, choroidal retinal neoplasias (**Figure 1B**). The height of the lesion reduced to 0.75 mm 18 months after crizotinib therapy by ultrasonography (**Figure 1C**). Examination by dilated funduscopy showed an amelanotic choroidal mass around the macula of the left eye consisting with a non-primitive choroidal neoplasm (**Figures 1D, E**). Chest CT revealed a nodule with hyperdense and a height of 15 mm in the posterior segment of the right upper lobe (**Figure 1F**), with enlargement of a single right mediastinal lymph node. Regression of the primary lesion 2 month after therapy (**Figure 1G**) and no progression of malignancy in the 18th month (**Figure 1H**).

**Figure 2:**
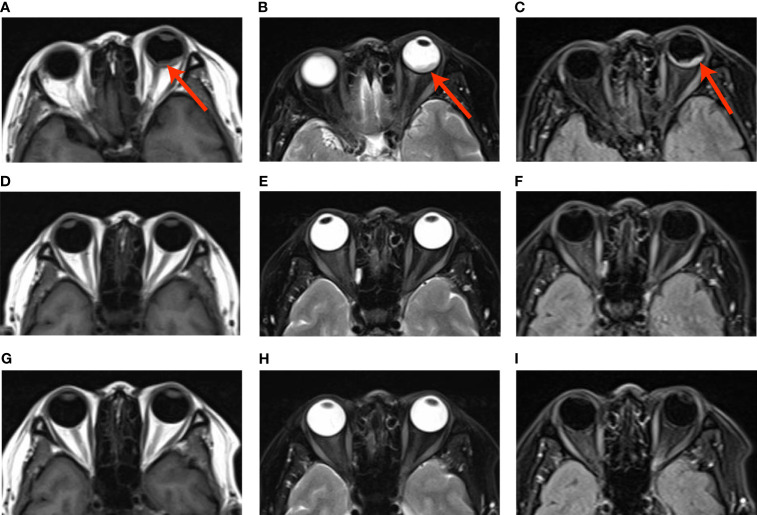
**(A–C)** Basal MRI-scan of the eyes, **(D–F)** reduction of the abnormal tissue in the left eye in 6th month crizotinib theraphy; **(G–H)**. No progression of malignancy on the 14th month crizitinib therapy.

Brain MRI indicated an irregular lesion protruding into the posterior wall of the left eye (**Figures 2A–C**), which is a hyperintensity to vitreous in T1-weighted MR image, and a hypointensity to vitreous in T2-weighted MR image. Bone scintigraphy and abdominal CT showed no obvious abnormality. Routine laboratory tests were within the normal range except for a slight elevation of carcinoembryonic antigen (CEA) to 16.5ng/ml.

**Figure 3:**
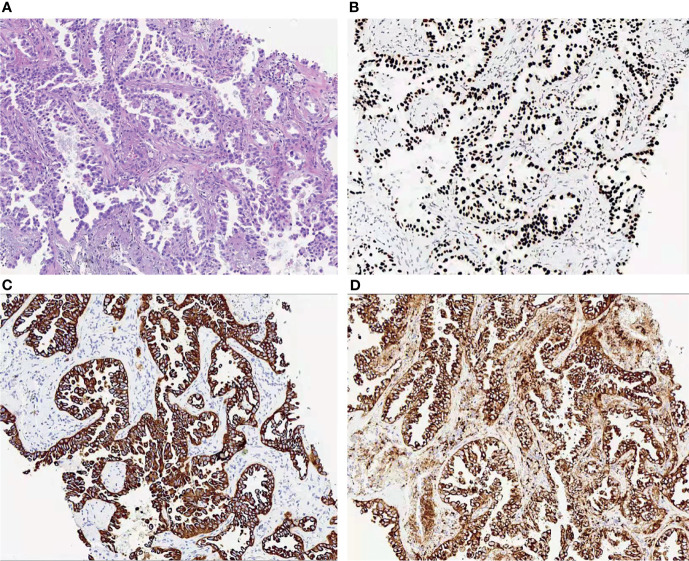
**(A)** Histopathological examination of the lung revealed a poor-differentiated adenocarcinoma; **(B–D)**. Immunohistochemical studies of the left lung showed to be positive for TTF-1 **(B)**, CK7 **(C)** and NapsinA **(D)**.

**Figure 4:**
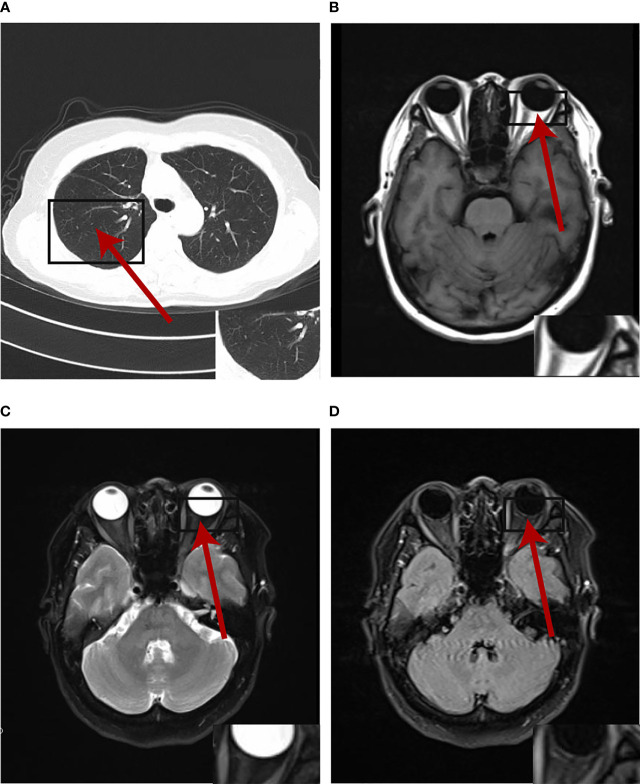
The primary lesion and oligogenous metastases were complete response by the patient's latest (August 2021) chest CT and cranial MRI at the time of writing.

The multi-disciplinary expert team includes ophthalmology, thoracic surgery, oncology, respirology, radiotherapy and radiology, reached a consensus on the treatment of this patient: removal of the right lung nodules followed by enucleation of the ailing eye. The patient underwent right superior lobe resection and lymph node dissection in April 2019. The resected specimen weighed 62g. A tumor located measured 3.4 × 2.5 × 2.0 cm in size. The cut surface revealed a well-encapsulated, solid tumor with a dark reddish color. The histopathological diagnosis was poor-differentiated infiltrating adenocarcinoma (solid type with mucinous+ acinar type). Bronchial terminus, pleural involvement, vascular cancer thrombosis, and spread through air space (STAS) were all negative. Lymph node involvement is as follows: “Group 2 lymph nodes” (0/1),” Group 3 lymph nodes “(0/1),” Group 4 lymph nodes” (0/1),” Group 5 lymph nodes “(0/2),” Group 7 lymph nodes” (0/5), “Group 9 lymph nodes” (0/1), “Group 10 lymph nodes” (5/6) and “Group 11 lymph nodes” (1/3). Immunohistochemical studies showed TTF-1, CK7 and NapsinA expression and the absence of CD56 (**Figure 3**). Postoperative pathology led to a diagnosis of right lung adenocarcinoma (T2aN1M1 stage), which was followed by next generation sequencing that revealed EML4-ALK (E13:A20) rearrangement. Because of the availability of targeted therapy, the patient declined enucleation of the eyeball which will impaired appearance. In view of the price difference, the patient chosed first-line treatment with crizotinib 250mg BID orally at the beginning of May 2019. Surprisingly, because of palliation of her photophobia, blurred vision and watering, the patient resumed normal daily activities after a 3-week course of crizotinib. Significant reduction of lesion in posterior wall of left eye was verified by comparison of basal MRI with MRI after 6 months of treatment, which showed regression of the previously observed lesions (**Figures 2D–F**). No progression in the 14th month crizitinib therapy by MRI (**Figures 2G–I**). The treatment had no side effects. The patient returned to the hospital every 3 months for evaluation of lesion and medication monitoring. At the time of this case report, the patient had been undergoing crizotinib therapy for over 27 months and remained stable with no systemic and ocular symptoms, confirmed by the patient’s latest (August 2021) chest CT and cranial MRI (**Figure 4**).

## Discussion

Ocular metastasis is rarely associated with lung cancer because the ophthalmic artery branches at right angles to the internal carotid artery, and tumor emboli in the blood are more likely to stay in the brain than enter the eye (3). The incidence of ocular metastasis in lung cancer is only about 0.1–7%, but the condition results in severe clinical symptoms (4).

The prevalence of symptomatic choroidal metastasis in cancer patients is approximately 1%–3%, whereas a prevalence of 9–12% has been reported from autopsy studies (5, 6). Although symptomatic choroidal metastasis may be rare in the initial presentation of lung cancer (7), patients with ocular symptoms require careful examination and detailed evaluation of their medical history. Similarly, when lung cancer patients present with blurred vision, eye pain and other ocular symptoms, oncologists should think about eye metastasis and a comprehensive eye examination should be arranged.

Cancer patients with metastasis traditionally have been regarded as representing a near terminal life stage and treated using only systemic therapy (8). For ocular metastases, local treatments are partial regression and remain limited, with the aim of maintaining visual function and improving quality of life, mainly by teletherapy, brachytherapy, intraocular injection of bevacizumab, photocoagulation, and even enucleation (9–12). Systemic treatment mainly includes chemotherapy, targeted therapy and immunotherapy. It is unclear whether the treatments described above are effective in oligometastases of NSCLC.

Here, for the first time, we document primary pulmonary resection in patients with lung adenocarcinoma with oligometastases. The primary 15-mm lesion was surgically removed and targeted therapy was continued, resulting in 27 months of progression-free survival (PFS) and a high quality of life. The concept of oligometastases was proposed by Hellman and Wechselbaum in 1995 in which the description of an intermediate state between localized cancer and wide-spread metastatic disease, termed ‘the oligometastatic state’, the treatment of oligometastatic disease (OMD) with curative intent has been gaining increasing acceptance (13, 14). The most important prognostic factor for oligometastases is the status of the primary lesion (15, 16). Therefore, we hypothesized that surgical resection of small primary lesions to reduce tumor load would be beneficial for patients with ocular oligometastases.

With improved survival of lung cancer patients treated with targeted therapies against driver mutations, the effect of choroidal metastases is unclear. ALK mutation in NSCLC is a rare subtype of oncogenic driver, accounting for approximately 5% of cases, whose the first-generation inhibitor, crizotinib (17–19), is widely used in patients and elicits a greater and longer response than chemotherapy.

To date only 9 ALK-rearranged NSCLC patients with choroidal metastasis who were treated with crizotinib/alectinib have been reported in the literature (**Table 1**). Most of them had been treated with radiation, chemotherapy, bevacizumab, and/or enucleation before anti-ALK therapy, with a best PFS of 18 months. Jiang et al. reported a patient who presented with choroidal metastasis in the left eye as the initial symptom of NSCLC and was eventually treated with enucleation and crizotinib after the ALK rearrangement was detected; however, this patient only had 6 months PFS (7). Fen et al. reported a patient who presented with choroidal metastasis in the left eye as the initial symptom of NSCLC and was treated with radiation and gefitinib before ALK 2p23 rearrangement was detected (20); the patient was eventually treated with crizotinib, which resulted in striking reductions in the symptoms after 10 months of therapy. Because of partial response of choroidal metastases after 3 weeks crizotinib treatment, the patient in the present report did not undergo the radiotherapy, chemotherapy or enucleation that had been scheduled to be performed after removal of the lung tumor. Up to now, she has had PFS for 27 months.

**Table 1:** Clinical data of 9 Reviewed lung cancer cases with eye metastasis.

NO	Age/sex/smoking status	OcularInvolvement	Onset with Ocular symptom	Histology	Primary lesion size	Metastatic Sites* (except eye)	Ocular Treatment/response	Systemic Treatment except TT/response	Target therapy	PFS of TT
**1**	44/M/N	LE	Y	ADC	LLL:55*40 mm	Liver, Cerebellum	RT/progression Enucleation	chemo(CV)+RT/PR chemo (D)/NA	Cirzotinib/PR	>6M
**2**	43/M/NA	BE	Y	ADC	RML	Bone	Surg/Regression	NO	Cirzotib/PR Alectinib/PR	15M >7M
**3**	31/M/N	BE	N	NSCLC	NA	Brain, Ribs	RT/NA	Chemo(platinum-based)+Bev +RT/PD	Erlotinib/NA Cirzotinib/PR	6M DWD
**4**	35/FE/N	RE	Y	ADC	LUL	Ribs acetabulum	NO	Chemo(cisplatin)+TT/PR	Cirzotinib/PR AP26113/SD	16M >10WK
**5**	62/M/NA	RE	Y	ADC	LUL:14mm 16mm	Pleura, Liver Adrenalin	NO	NO	Alectinib/PR	NA
**6**	62/M/NA	LE	N	ADC	BL:36*27mm	Liver, Bone,	NO	NO	Alectinib/PR	>2M
**7**	30/FE/N	LE	Y	NSCLC	NA	Liver, Bone	NO	Anti-PD-1 Antibody chemo(AP)/PR	Crizotinib/PR Alectinib/PR	6M >5M
**8**	53/FE/N	LE	N	ADC Breast Carcinoma	NA	Pleura, Bone Liver	RT	Chemo(PC)+Bev/PD Chemo(D)+ nintedanib/PD Chemo(AP)/PD	Crizotinib/PD	10M
**9**	40/FE/Y	RE	Y	ADC	LUL	Liver Bone	NO	Chemo(AP) +Bev/PR	Alectinib/PR	9M

ADC, adenocarcinoma; AP, pemetrexed-cisplatin; BE, bilateral eyes; Bev, bevacizumab; BL, bilateral lung; Chemo, chemotherapy; CTx-NS, chemotherapy (not specified); CV, cisplatin-vinorelbine; D, Docetaxel; FE, female; LE, left eye; LLL, left lower lobe; LUL, left upper lobe; M, male; N, no; NSCLC, non-small cell lung carcinoma; NA, data not available/not presented; RE, right eye; PC, Paclitaxel-carboplatin; PD, progressive disease; PR, partial response; RML, right middle lobe; RT, radiation therapy; SD, Stable disease; Surg, surgery; TT, Target therapy; Y, yes; DWD, Died with disease.

Targeted therapy is playing a significant role in the treatment of lung cancer, but drug resistance is inescapable which is more intractable and intensifies the psychological burden on patients (21, 22). In crizotinib-resistant cases, patient switched to alectinib observed a 55% overall response rate (23, 24). Two studies reported that patients who are resistant to crizotinib as the first-line treatment achieved significant PFS when they were switched to aletinib. Okuma et al. reported a 30-year-old female patient who had choroidal metastasis of crizotinib-resistant ALK-rearranged NSCLC who was successfully treated with alectinib (25). The patient underwent first-line crizotinib, chemotherapy and third-line treatment with the PD-1 (programmed cell death-1) targeted therapy. At the beginning of third-line treatment, who was rebiopsied due to the progress of liver. Two PD-1 targeted therapy weeks later, systemic symptoms, including fever, bone pains, arthralgia, and visual disturbance were exacerbated. She started alectinib treatment and both her ocular and systemic symptoms were palliated in a week. Bearz.A et al.reported the case of a patient with choroidal metastasis in progression in the right lung and mediastinal lymph nodes after 15 months of crizotinib treatment, and then switched to oral aletinib and went into remission after 7 months (26).

## Conclusion

We reported an ALK-rearranged NSCLC patient with oligogenous metastases and initial ocular symptoms who underwent surgery to remove the primary lesion followed by targeted therapy. The patient achieved a durable response to crizotinib without systemic chemotherapy, radiotherapy or enucleation. At the best of our knowledge, she has had the longest PFS of any patient with known lung cancer and ocular metastases who was treated with crizotinib, 27 months as of this writing. Based on this, oncologists and ophthalmologists be aware of this kind of presentation as early initiation of therapy is important to preserve vision, and resection of the lung lesions is feasible therapeutic strategies. In addition, it is important to confirm the mechanism of the primary cancer and identify any oncogene mutations as soon as possible to get effective therapies (27).

## Data Availability Statement

The original contributions presented in the study are included in the article/supplementary material. Further inquiries can be directed to the corresponding author.

## Ethics Statement

Written informed consent was obtained from the individual(s) for the publication of any potentially identifiable images or data included in this article.

## Author Contributions

SL and TW were the attending physicians of the patient, responsible for case collection and article writing. XL, XY, WL, and QY followed up the patient and arrange examinations. CZ and BR reviewed literatures and retrieved relevant patient information. MX and CY collated the data. All authors contributed to the article and approved the submitted version.

## Funding

The Cost of Publication was provided by the Research Program of Chengdu University of Chinese Traditional Medicine(NO. YYZX2020014).

## Conflict of Interest

The authors declare that the research was conducted in the absence of any commercial or financial relationships that could be construed as a potential conflict of interest.

## Publisher’s Note

All claims expressed in this article are solely those of the authors and do not necessarily represent those of their affiliated organizations, or those of the publisher, the editors and the reviewers. Any product that may be evaluated in this article, or claim that may be made by its manufacturer, is not guaranteed or endorsed by the publisher.
